# Assessment of ventilation heterogeneity in severe asthma using phase‐resolved functional lung magnetic resonance imaging

**DOI:** 10.14814/phy2.70423

**Published:** 2025-06-18

**Authors:** Chuan T. Foo, David Langton, Graham M. Donovan, Bruce R. Thompson, Peter B. Noble, Francis Thien

**Affiliations:** ^1^ Faculty of Medicine, Nursing and Health Sciences Monash University Melbourne Victoria Australia; ^2^ Department of Respiratory Medicine Eastern Health Melbourne Victoria Australia; ^3^ Department of Thoracic Medicine Peninsula Health Frankston Victoria Australia; ^4^ Department of Mathematics University of Auckland Auckland New Zealand; ^5^ School of Health Sciences University of Melbourne Melbourne Victoria Australia; ^6^ School of Human Sciences The University of Western Australia Perth Western Australia Australia

**Keywords:** asthma, functional lung imaging, lung physiology, magnetic resonance imaging, ventilation heterogeneity

## Abstract

Ventilation heterogeneity is a hallmark of asthma. This study examines the feasibility of phase‐resolved functional lung magnetic resonance imaging (PREFUL MRI) in the evaluation of ventilation heterogeneity in severe asthma, its response to bronchodilator, and correlation with spirometry. Twenty‐three patients with severe asthma and seven healthy volunteers completed PREFUL MRI and spirometry pre and post‐bronchodilator. Ventilation heterogeneity was assessed using ventilation defect percentages (VDP) for regional ventilation (RVent) and flow‐volume loop cross‐correlation (FVL), interquartile distance (IQD), and inhomogeneity index (IHI). Patients exhibited a significantly higher pre‐bronchodilator VDP_RVent_ (19.9 ± 14.0 vs. 1.9 ± 1.9%, *p* < 0.001), VDP_FVL_ (21.6 ± 15.9 vs. 1.7 ± 2.1%, *p* < 0.001), IQD (0.60 ± 0.25 vs. 0.30 ± 0.06, *p* < 0.001), and IHI (0.34 ± 0.12 vs. 0.18 ± 0.04, *p* < 0.001) compared to healthy volunteers. Post‐bronchodilator, VDP_RVent_ (14.7 ± 12.5 vs. 19.9 ± 14.0%, *p* = 0.02), IQD (0.51 ± 0.20 vs. 0.60 ± 0.25, *p* = 0.02), and IHI (0.30 ± 0.11 vs. 0.34 ± 0.12, *p* = 0.02) decreased significantly in patients but remained significantly higher than in healthy volunteers. Significant correlations were observed between pre‐bronchodilator FEV1 and VDP_RVent_ (*ρ* = −0.61, *p* < 0.001), VDP_FVL_ (*ρ* = −0.73, *p* < 0.001), IQD (*ρ* = −0.57, *p* = <0.001), and IHI (*ρ* = −0.60, *p* < 0.001). PREFUL MRI derived markers of ventilation heterogeneity are worse in patients with asthma, improve post‐bronchodilator, and correlate with the severity of airflow obstruction. These findings support the role of PREFUL MRI in assessing ventilation heterogeneity in asthma.

## Graphical abstract

Graphical abstract showing some of the PREFUL‐derived ventilation biomarkers used in this study (left panel) and their response to bronchodilator therapy (top right panel) as well as correlations with FEV1 (bottom right panel). FEV1, forced expiratory volume in 1 s; HV, healthy volunteer; IQD, interquartile distance; PostBD, post‐bronchodilator; PreBD, pre‐bronchodilator; PT, patients with severe asthma; *ρ*, Spearman rho; VDP_RVent_, regional ventilation defect percentage; VDP_FVL_, flow volume loop cross‐correlation ventilation defect percentage.

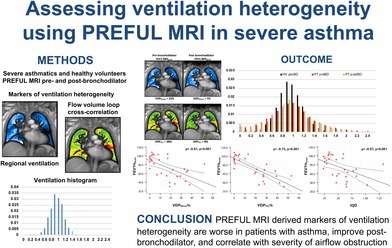

## 1 INTRODUCTION

Asthma is a common disease that affects approximately 1 in 9 individuals worldwide and contributes greatly to the global disease burden (Global Burden of Disease Collaborative Network, 2020). Asthma is characterized by airway inflammation, hyper‐responsiveness, variable airway obstruction, and manifests clinically as cough, chest tightness, wheeze, and shortness of breath (Varricchi et al., 2024). Ventilation heterogeneity, which refers to the nonuniform distribution of inspired gas within the lungs, is a hallmark feature of asthma and a determinant of poor asthma control (Gibson et al., 2024; King et al., 2019; Kraft et al., 2022; Svenningsen et al., 2018; Tang et al., 2020). Possible causes of ventilation heterogeneity in asthma include airway narrowing due to bronchoconstriction, inflammation, or mucus plugs; dynamic airway closure; and airway remodeling (Costella et al., 2012; Downie et al., 2013; Gerald Teague et al., 2021; Nilsen et al., 2019; Samee et al., 2003; Svenningsen et al., 2018).

Over the years, advances in functional lung imaging have evolved to include several modalities for assessing lung ventilation. Hyperpolarized gas magnetic resonance imaging (MRI), the most established of these modalities, is commonly regarded as the gold standard, although its use is limited to specialized academic institutions due to high upfront cost, complex imaging pipeline, and need for skilled personnel (Foo et al., 2023). Alternatives such as single‐photon emission computed tomography and ventilation‐perfusion scintigraphy suffer from poor spatial resolution, long acquisition times, and need for ionizing radiation (Bourhis et al., 2020). CT‐based techniques like Xenon‐CT (Kong et al., 2014) and Functional Respiratory Imaging (De Backer et al., 2010) are at different stages of maturity, involve high doses of radiation, and sophisticated image acquisition and post‐processing.

Phase‐resolved functional lung magnetic resonance imaging (PREFUL MRI) is a relatively new imaging modality that utilizes the changes in endogenous lung magnetic resonance signal during tidal respiration as a proxy for lung ventilation and perfusion (Voskrebenzev et al., 2018). The advantages of PREFUL MRI include the lack of ionizing radiation, low setup cost (PREFUL MRI can be performed on any standard clinical MRI scanner without the requirement for additional costly equipment), short scanning time, and no need for breath‐holding maneuvers. Commonly derived semiquantitative functional parameters include regional ventilation, flow‐volume loop cross‐correlation, normalized and quantified perfusion, and their respective defect percentages (Pohler et al., 2021; Voskrebenzev et al., 2018).

To date, PREFUL MRI has been successfully applied in a variety of patient populations—chronic obstructive pulmonary disease (Kaireit et al., 2021; Pohler et al., 2021; Vogel‐Claussen et al., 2019; Voskrebenzev et al., 2022), cystic fibrosis (Couch et al., 2021; Dohna et al., 2024; Marshall et al., 2023; Munidasa et al., 2021), chronic lung allograft dysfunction (Moher Alsady et al., 2019; Vogel‐Claussen et al., 2023), COVID‐19 (Levy et al., 2022; Wang et al., 2022), and chronic thromboembolic pulmonary hypertension (Moher Alsady, Voskrebenzev, et al., 2024; Pohler et al., 2020). These studies have shown PREFUL MRI parameters to be sensitive to treatment (Scheller et al., 2024; Dohna et al., 2024; Friedlander et al., 2024; Munidasa et al., 2021; Vogel‐Claussen et al., 2019; Voskrebenzev et al., 2022) and to have moderate correlation with lung function tests (Couch et al., 2021; Dohna et al., 2024; Friedlander et al., 2024; Kaireit et al., 2021; Marshall et al., 2023; Munidasa et al., 2021; Voskrebenzev et al., 2022) and other imaging modalities including hyperpolarized ^129^Xe MRI (Couch et al., 2021; Friedlander et al., 2024; Kaireit et al., 2021; Marshall et al., 2023; Munidasa et al., 2021). Despite these promising findings, there are limited data on the utility of PREFUL MRI in severe asthma, with only one prior study identified (Friedlander et al., 2024). In that study, PREFUL MRI derived ventilation defect percentage (VDP) was used to compare responsiveness to bronchodilator therapy in severe asthma patients and healthy controls. To our knowledge, the use of PREFUL MRI to evaluate ventilation heterogeneity using metrics other than VDP has not been reported.

In this study, we aimed to investigate if PREFUL MRI can be used to (i) compare differences in ventilation heterogeneity between healthy volunteers and patients with severe asthma; (ii) assess response to bronchodilator therapy; (iii) evaluate ventilation heterogeneity using metrics other than VDP; and (iv) explore the relationships between these measurements and established clinical physiological parameters such as spirometry and plethysmographic lung function tests.

## 2 METHODS

### 2.1 Participants and study design

This prospective study involved patients with severe asthma and healthy volunteers who completed research assessments at two Australian university teaching hospitals. Participants were required to undergo lung function tests and PREFUL MRI before and after bronchodilator over two separate visits no longer than 5 days apart. The study was approved by the Eastern Health Human Research Ethics Committee (E21‐021‐79977) and all participants provided written informed consent.

Patients recruited required a diagnosis of severe asthma as defined by the European Respiratory Society/American Thoracic Society (ERS/ATS) (Chung et al., 2014). Specifically, all patients needed to show evidence of uncontrolled asthma, such as a high symptom burden or frequent asthma exacerbations in the preceding 12 months despite receiving Global Initiative for Asthma (GINA) Step 4–5 treatment (Global Initiative for Asthma, 2024). Healthy volunteers were required to be never smokers with no history of any health conditions.

Exclusion criteria included (i) participants with an alternative respiratory condition such as chronic obstructive pulmonary disease, bronchiectasis, or interstitial lung disease, (ii) females who were pregnant or lactating, (iii) participants aged less than 18 years, or who were unable to provide written informed consent, and (iv) participants unable or unwilling to undergo MRI imaging.

### 2.2 Image acquisition and analysis

MRI scans were acquired using a 3.0 Tesla MRI scanner (Skyra, Siemens Healthineers, Erlangen, Germany) with an 18 channel body array coil. A spoiled gradient echo sequence was used to acquire 250 single‐slice coronal images centered on the carina. The following scan parameters were used: field of view 500 × 500 mm^2^, matrix size 128 × 128, slice thickness 15 mm, echo time 0.83 msec, repetition time 2.1 msec, bandwidth 1860 Hz/pixel, flip angle 4°, and a total scan duration of 35 s at a temporal resolution of 135 msec/frame. Image acquisition occurred during tidal respiration. All participants were imaged at baseline and post bronchodilator (400 μg salbutamol).

Post‐acquisition, MRI images were analyzed using a graphical user interface application developed by Voskrebenzev et al. (Biovisioneers, 2025). The application mimics the PREFUL analysis pipeline originally proposed by Voskrebenzev et al. (2018), and performs the necessary image registration, segmentation, filtering, phase sorting, and biomarker extraction. In brief, all images were registered to a reference image in an intermediate respiratory position using Advanced Normalization Tools with a group‐orientated registration approach (Voskrebenzev et al., 2017). Lung segmentation was performed by an automated convolutional neural network (Crisosto et al., 2023) with vessels removed using Otsu methods for thresholding (Otsu, 1979). Manual adjustments were made if necessary. For the ventilation analysis, a low pass filter was first applied to remove signal variations due to perfusion. Next, images were sorted according to their phase by analyzing a spatially averaged lung signal with a cosine model function to create one respiratory cycle with increased temporal resolution (Voskrebenzev et al., 2018). Regional ventilation (RVent) was calculated for all voxel within the lung mask as per the following equation (Klimes et al., 2019),

$$\text{RVent}\;=\frac{S_{\mathrm{Mid}}}{S_{\text{Insp}}}-\frac{S_{\mathrm{Mid}}}{S_{\mathrm{Exp}}}$$

where *S*_Mid_, *S*_Insp_, and *S*_Exp_ represent the MR signal at the midway between inspiration and expiration, at the end‐inspiratory state, and the end‐expiratory state, respectively. Using a stochastically driven adaptive threshold, the normal range of RVent was defined as the 90th percentile of the cumulative signal intensity distribution of the lung mask multiplied by an empirically chosen factor of 0.4 (Pohler et al., 2021). Conversely, the RVent VDP was calculated as the percent of the lung/voxels in the slice being analyzed that falls below this threshold.

Using data from the entire respiratory cycle, flow volume loops were also generated for all voxels within the lung mask and correlated to a healthy reference flow volume loop by means of cross‐correlation (Moher Alsady et al., 2019). The healthy reference flow volume loop was determined by averaging the individual flow volume loops in a selected region of the lung containing RVent values within the 75th–95th percentile range, thus excluding regions with either hypoventilation or excessively high ventilation (Moher Alsady et al., 2019; Pohler et al., 2021). This resulted in a flow volume loop cross correlation (FVL) map in percent, with 100% indicating perfect correlation with the healthy reference, and 0% indicating no agreement with the healthy reference. Values ≥90% were defined as normal, with the FVL VDP referring to the percent of the lung/voxels in the slice being analyzed that exhibits a <90% correlation with the healthy reference (Pohler et al., 2021).

Additionally, masked PREFUL ventilation images were processed using MATLAB (R2023b, MathWorks, Natick, Massachusetts, USA) to calculate the voxel‐value histograms after excluding extreme outliers (defined as values above the 99th percentile). The differences in the shape and position of the histograms provide information on the changes in ventilation distribution before and after bronchodilator administration, and between participant groups. Several heterogeneity parameters including skewness, kurtosis, and interquartile distance (IQD; defined as the interquartile range divided by the mean), and inhomogeneity index (IHI) were also calculated. The latter is a parameter commonly used to quantify lung ventilation heterogeneity during tidal respiration in electrical impedance tomography, and is calculated using the following formula (Heines et al., 2022; Zhao et al., 2009) where *RVent*_*xy*_ represents the RVent value of individual pixels (x,y) within the lung mask, and Median RVent_lung_ represents the median of the RVent values within the lung mask.

$$\mathrm{IHI}=\frac{\sum_{x,y\in \text{lung}}\left|{\text{RVent}}_{\mathrm{xy}}-\text{Median}\;\left({\text{RVent}}_{\text{lung}}\right)\right|}{\sum_{x,y\in \text{lung}}{\text{RVent}}_{\mathrm{xy}}}$$

Together, these parameters provide information on ventilation heterogeneity.

### 2.3 Lung function tests

All participants performed pre‐ and post‐bronchodilator spirometry, with patients withholding their usual bronchodilators for 12–24 h prior to testing. Pre‐bronchodilator plethysmographic lung volumes were also measured in patients. All tests were performed using the MGC Diagnostics Platinum Elite (Minnesota, USA) and conducted in accordance with ERS/ATS standards (Graham et al., 2019). Predicted value equations were taken from the Global Lung Initiative (Hall et al., 2021; Quanjer et al., 2012; Stanojevic et al., 2017).

### 2.4 Statistical analysis

Normality assessment was performed for all continuous variables using the Shapiro–Wilk test. Distributions were summarized using mean, median, standard deviation, or interquartile range. Categorical variables were presented as number and percentage.

Between‐group comparisons were performed using *χ* ^2^ or Fisher's exact test for categorial variables and Student's *t*‐test or Mann–Whitney *U*‐test for continuous variables. Paired group measurements were determined using paired *t*‐test or Wilcoxon signed‐rank test. Relationships between variables were assessed using Spearman correlation with the Hochberg method used to adjust for multiple comparisons. Statistical analyses were performed using SPSS Version 29 (IBM corporation, New York, USA). A *p* value of <0.05 was considered statistically significant.

## 3 RESULTS

### 3.1 Baseline characteristics

Twenty‐three patients (5 males and 18 females) with severe asthma and seven healthy volunteers (all females) were included in this study. All participants completed research assessments as described. Patients were significantly older (51.9 ± 17.6 vs. 25.7 ± 5.4 years, *p* < 0.001), and had higher BMI (31.8 ± 6.9 vs. 23.0 ± 2.6 kg/m^2^, *p* = 0.003) than healthy volunteers. None of the patients were active smokers—14 never smokers; 8 with a <5 packet year history; and 1 with a >5 packet year history. All healthy volunteers were never smokers by definition. Patients were highly symptomatic with a mean ACQ of 3.0 ± 1.0 despite high‐dose inhaled corticosteroids with beclomethasone‐equivalent dose 1930 ± 839 μg/day. Fifteen patients were also on additional GINA Step 5 therapies including 6 (26.1%) patients receiving maintenance oral prednisolone with a group median dose of 10 (10–40) mg/day, and 9 (39.1%) patients on an asthma biologic. These are summarized in Table 1.

**TABLE 1:** Baseline characteristics.

	Patients (*n* = 23)	Healthy volunteers (*n* = 7)	*p* Value
Age (years)	51.9 ± 17.6	25.7 ± 5.4	<0.001
Males/Females	5 (22)/18 (78)	0 (0)/7 (100)	0.3
BMI (kg/m^2^)	31.8 ± 6.9	23.0 ± 2.6	0.003
Smoking status			
Never smoker	14 (61)	7 (100)	0.07
Ex‐smoker	9 (39)	0 (0)	
ACQ	3.0 ± 1.0	–	
Beclomethasone‐equivalent dose (μg/day)	1930 ± 839	–	
Other therapies			
Maintenance OCS	6 (26)	–	
Asthma biologic	9 (39)		

*Note*: Values are presented as mean ± standard deviation or number (%).

Abbreviations: ACQ, asthma control questionnaire; BMI, body mass index; OCS, oral corticosteroid.

### 3.2 Spirometry

Patients had a significantly lower baseline FEV1%_pred_ (68.9 ± 24.5 vs. 92.0 ± 12.5%, *p* = 0.003) and forced expiratory ratio (FER, ratio of FEV1 to FVC; 58.8 ± 26.2 vs. 84.7 ± 7.30%, *p* < 0.001) when compared to healthy volunteers. Post‐bronchodilator, patients showed significant improvements in FEV1%_pred_ (77.9 ± 20.8 vs. 68.9 ± 24.5%, p < 0.001), FVC%_pred_ (90.8 ± 15.3 vs. 84.1 ± 18.4%, *p* < 0.001), and FER (62.9 ± 28.5 vs. 58.8 ± 26.2, *p* = 0.03). Although FEV1%_pred_ (95 ± 13.1 vs. 92.0 ± 12.5%, *p* = 0.003) and FER (88.0 ± 6.1 vs. 84.7 ± 7.3, *p* = 0.01) also significantly increased post‐bronchodilator in healthy volunteers, the change was clinically insignificant (see Table 2).

**TABLE 2:** Spirometry.

Patients vs. healthy volunteers—pre‐bronchodilator			
Variable	Patients	Healthy volunteer	*p* Value
FEV1%_pred_	68.9 ± 24.5	92.0 ± 12.5	0.003
FVC%_pred_	84.1 ± 18.4	94.7 ± 17.2	0.19
FER	58.8 ± 26.2	84.7 ± 7.3	<0.001

Patients—pre‐ and post‐bronchodilator			
Variable	Pre‐bronchodilator	Post‐bronchodilator	*p* Value
FEV1%_pred_	68.9 ± 24.5	77.9 ± 20.8	<0.001
FVC%_pred_	84.1 ± 18.4	90.8 ± 15.3	<0.001
FER	58.8 ± 26.2	62.9 ± 28.5	0.03

Healthy volunteers—pre‐ and post‐bronchodilator			
Variable	Pre‐bronchodilator	Post‐bronchodilator	*p* Value
FEV1%_pred_	92.0 ± 12.5	95 ± 13.1	0.003
FVC%_pred_	94.7 ± 17.2	94 ± 16.2	0.18
FER	84.7 ± 7.3	88.0 ± 6.1	0.01

Abbreviations: FER, forced expiratory ratio; FEV1, forced expiratory volume in 1 s; FVC, forced vital capacity.

### 3.3 Ventilation and ventilation heterogeneity

**FIGURE 1:**
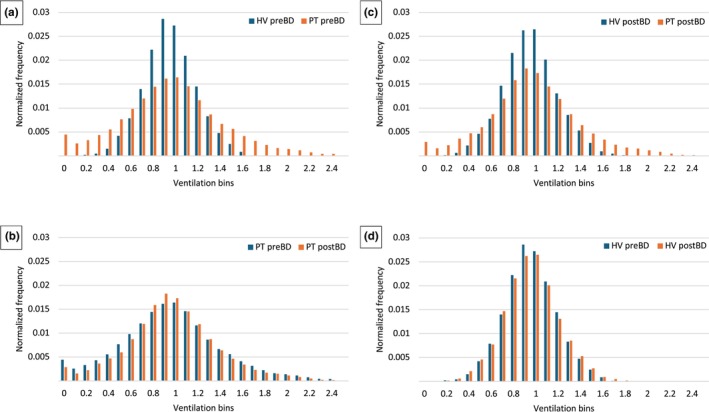
Pooled normalized ventilation histograms between patients and healthy volunteers pre‐bronchodilator (a), post‐bronchodilator (c), and between patients pre‐ and post‐bronchodilator (b), and healthy volunteers pre‐ and post‐bronchodilator (d). (a and c) Patients show a more heterogeneous distribution of ventilation compared to healthy volunteers both pre‐ and post‐bronchodilator. (b) Patients show a reduction in ventilation heterogeneity post‐bronchodilator represented by a shift in the frequency distribution of ventilation from the lower and higher ends towards the center. (d) Healthy volunteers appeared to show an increase in ventilation heterogeneity post‐bronchodilator. HV, healthy volunteer; PostBD, post‐bronchodilator; PreBD, pre‐bronchodilator; PT, patients with severe asthma.

At baseline, patients with asthma had a significantly higher VDP_RVent_ (19.9 ± 14.0 vs. 1.9 ± 1.9%, *p* < 0.001), VDP_FVL_ (21.6 ± 15.9 vs. 1.7 ± 2.1%, *p* < 0.001), IQD (0.60 ± 0.25 vs. 0.30 ± 0.06, *p* < 0.001), and IHI (0.34 ± 0.12 vs. 0.18 ± 0.04, *p* < 0.001) than healthy volunteers (Table 3 and Figure 1a).

**TABLE 3:** Comparison of ventilation biomarkers in patients and healthy volunteers.

Patients vs. healthy volunteers—pre‐bronchodilator			
Variable	Patients	Healthy volunteer	*p* Value
VDP_RVent_ (%)	19.9 ± 14.0	1.9 ± 1.9	<0.001
VDP_FVL_ (%)	21.6 ± 15.9	1.7 ± 2.1	<0.001
IQD	0.60 ± 0.25	0.30 ± 0.06	<0.001
IHI	0.34 ± 0.12	0.18 ± 0.04	<0.001
Kurtosis	2.82 ± 0.65	2.87 ± 0.30	0.87
Skewness	0.22 ± 0.37	0.13 ± 0.23	0.58

Patients vs. healthy volunteers—post‐bronchodilator			
Variable	Patients	Healthy volunteer	*p* Value
VDP_RVent_ (%)	14.7 ± 12.5	2.3 ± 2.4	<0.001
VDP_FVL_ (%)	18.1 ± 18.0	1.0 ± 1.2	<0.001
IQD	0.51 ± 0.20	0.31 ± 0.06	<0.001
IHI	0.30 ± 0.11	0.19 ± 0.03	<0.001
Kurtosis	3.22 ± 1.28	2.90 ± 0.15	0.53
Skewness	0.34 ± 0.40	0.15 ± 0.17	0.25

Patients—pre‐ vs. post‐bronchodilator			
Variable	Pre‐bronchodilator	Post‐bronchodilator	*p* Value
VDP_RVent_ (%)	19.9 ± 14.0	14.7 ± 12.5	0.02
VDP_FVL_ (%)	21.6 ± 15.9	18.1 ± 18.0	0.31
IQD	0.60 ± 0.25	0.51 ± 0.20	0.02
IHI	0.34 ± 0.12	0.30 ± 0.11	0.02
Kurtosis	2.82 ± 0.65	3.22 ± 1.28	0.18
Skewness	0.22 ± 0.37	0.34 ± 0.40	0.18

Healthy volunteers—pre‐ vs. post‐bronchodilator			
Variable	Pre‐bronchodilator	Post‐bronchodilator	*p* Value
VDP_RVent_ (%)	1.9 ± 1.9	2.3 ± 2.4	0.48
VDP_FVL_ (%)	1.7 ± 2.1	1.0 ± 1.2	0.25
IQD	0.30 ± 0.06	0.31 ± 0.06	0.29
IHI	0.18 ± 0.04	0.19 ± 0.03	0.09
Kurtosis	2.87 ± 0.30	2.90 ± 0.15	0.74
Skewness	0.13 ± 0.23	0.15 ± 0.17	0.83

Abbreviations: IHI, inhomogeneity index; IQD, interquartile distance; VDP_FVL_, flow volume loop cross‐correlation ventilation defect percentage; VDP_RVent_, regional ventilation defect percentage.

Post bronchodilator, patients with asthma exhibited a significant reduction in VDP_RVent_ (14.7 ± 12.5 vs. 19.9 ± 14.0%, *p* = 0.02), IQD (0.51 ± 0.20 vs. 0.60 ± 0.25, *p* = 0.02), and IHI (0.30 ± 0.11 vs. 0.34 ± 0.12, p = 0.02) but not VDP_FVL_ (18.1 ± 18.0 vs. 21.6 ± 15.9%, *p* = 0.31). (Table 3 and Figure 1b).

Nonetheless, when compared to healthy volunteers post‐bronchodilator, patients with asthma still demonstrated a significantly elevated post‐bronchodilator VDP_RVent_ (14.7 ± 12.5 vs. 2.3 ± 2.4%, *p* < 0.001), VDP_FVL_ (18.1 ± 18.0 vs. 1.0 ± 1.2%, *p* < 0.001), IQD (0.51 ± 0.20 vs. 0.31 ± 0.06, *p* < 0.001), and IHI (0.30 ± 0.11 vs. 0.19 ± 0.03, *p* < 0.001) (Table 3 and Figure 1c).

Bronchodilator did not have any significant effect on any of these variables in healthy volunteers although the pooled normalized histogram of the ventilation distribution appeared to suggest an increase in ventilation heterogeneity (Table 3 and Figure 1d). No significant difference was observed in kurtosis or skewness of ventilation distribution within or between groups.

**FIGURE 2:**
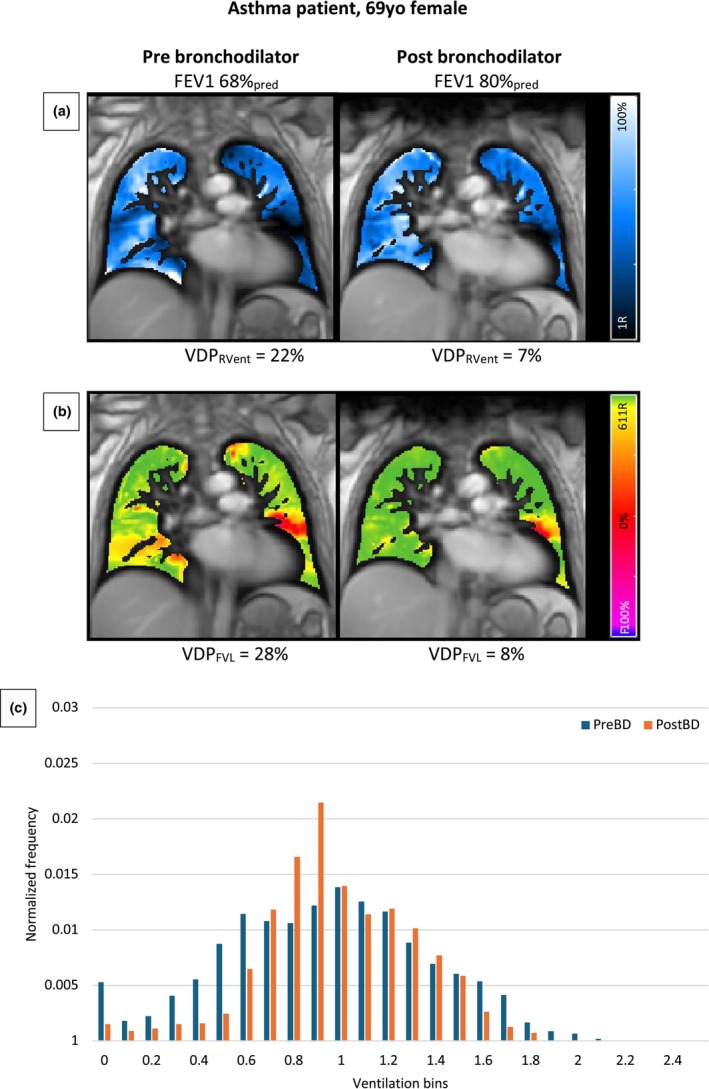
PREFUL regional ventilation (a) and flow volume loop cross‐correlation maps (b) pre‐ and post‐bronchodilator in a 69‐year‐old female with severe asthma. The images show a high ventilation defect burden at baseline, which improves after bronchodilator therapy. Normalized ventilation histogram (c) of the same patient showing a reduction in ventilation heterogeneity post‐bronchodilator represented by a shift in the frequency distribution of ventilation from the lower and higher ends towards the center. The patient has provided written informed consent for reproduction of this image. FEV1, forced expiratory volume in 1 s; PostBD, post‐bronchodilator; PreBD, pre‐bronchodilator; VDP_FVL_, flow volume loop cross‐correlation ventilation defect percentage; VDP_RVent_, regional ventilation defect percentage.

**FIGURE 3:**
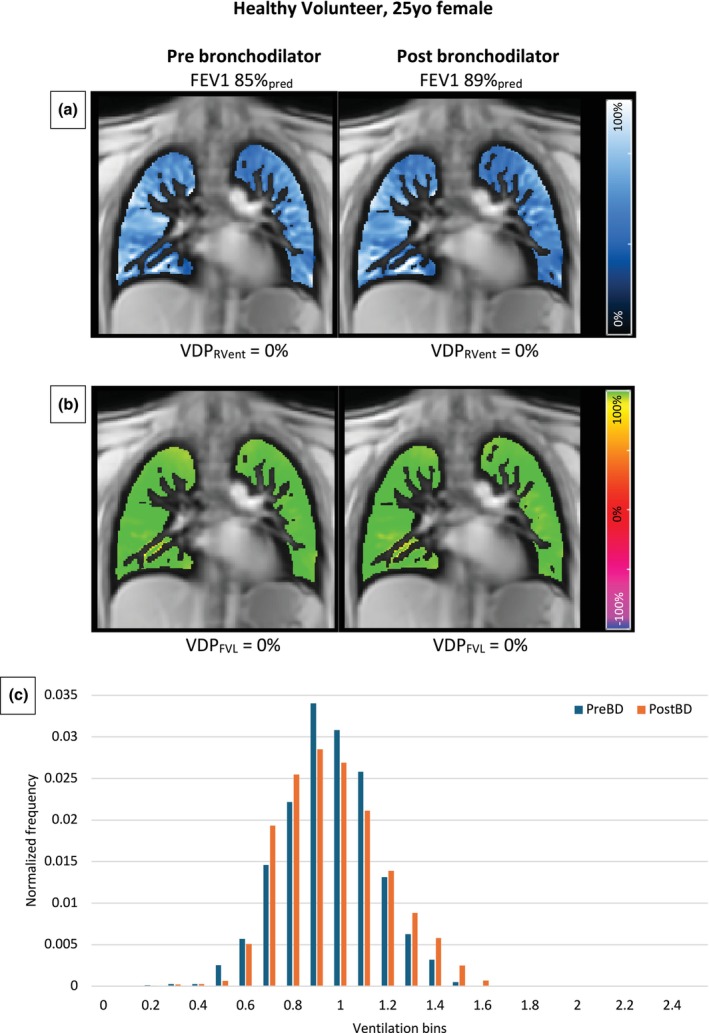
PREFUL regional ventilation (a) and flow volume loop cross‐correlation maps (b) pre‐ and post‐bronchodilator in a 25‐year‐old female healthy volunteer. The images show relatively homogeneous ventilation with no significant change post‐bronchodilator. Normalized ventilation histogram (c) of the same patient before and after bronchodilator. The patient has provided written informed consent for reproduction of this image. FEV1, forced expiratory volume in 1 s; PostBD, post‐bronchodilator; PreBD, pre‐bronchodilator; VDP_FVL_, flow volume loop cross‐correlation ventilation defect percentage; VDP_RVent_, regional ventilation defect percentage.

Figures 2 and 3 show coronal PREFUL regional ventilation, flow volume loop cross‐correlation maps and normalized ventilation histogram for a representative patient with asthma and a healthy volunteer. In the healthy volunteer, a relatively homogeneous distribution can be seen in the regional ventilation and flow volume loop cross‐correlation maps, with minimal change after bronchodilator administration. In contrast, these maps show a more heterogeneous distribution with a greater defect burden in the patient with asthma, along with a significant improvement post‐bronchodilator. Similarly, the normalized ventilation histograms depict a greater reduction in ventilation heterogeneity post‐bronchodilator in the patient with asthma compared to the healthy volunteer.

### 3.4 Correlations

**FIGURE 4:**
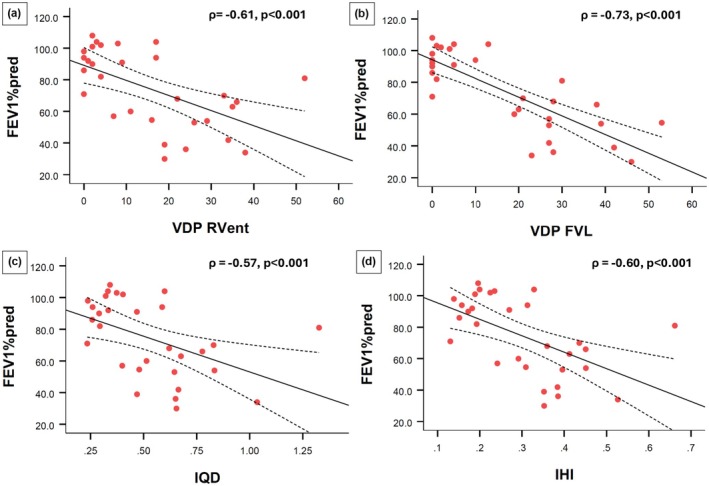
Relationships between FEV1%_pred_ and PREFUL derived biomarkers. Correlation between FEV1%_pred_ and (a) VDP_RVent_% (*ρ* = −0.61, *p* < 0.001), (b) VDP_FVL_% (*ρ* = −0.73, *p* < 0.001), (c) IQD (*ρ* = −0.57, *p* < 0.001), and (d) IHI (*ρ* = −0.60, *p* < 0.001). All plots show data for all participants (healthy volunteers and patients with asthma) pre‐bronchodilator. The solid line represents the regression line and the dashed lines show the 95% confidence bands. FEV1, forced expiratory volume in 1 s; IHI, inhomogeneity index; IQD, interquartile distance; VDP_FVL_, flow volume loop cross‐correlation ventilation defect percentage; VDP_RVent_, regional ventilation defect percentage; *ρ*, Spearman rho.

The relationships between lung function and PREFUL‐derived ventilation biomarkers were also explored (Table 4). Pre‐bronchodilator, significant correlations were found between FEV1%_pred_ and baseline VDP_RVent_ (*ρ* = −0.61, *p* < 0.001), VDP_FVL_ (*ρ* = −0.73, *p* < 0.001), IQD (*ρ* = −0.57, *p* < 0.001), and IHI (*ρ* = −0.60, *p* < 0.001) for all study participants (healthy volunteers and patients with asthma) (Figure 4); and between RV/TLC% and VDP_RVent_ (*ρ* = 0.50, *p* = 0.02), VDP_FVL_ (*ρ* = 0.70, *p* < 0.001), IQD (*ρ* = 0.43, *p* = 0.04), and IHI (*ρ* = 0.50, *p* = 0.02) for patients with asthma.

**TABLE 4:** Relationships between lung function and ventilation biomarkers.

	PreBD FEV1%_pred_		PostBD FEV1%_pred_		PreBD RV/TLC%	
	*ρ*	*p* Value	*ρ*	*p* Value	*ρ*	*p* Value
VDP_RVent_ (%)	−0.61	<0.001	−0.53	0.003	0.50	0.02
VDP_FVL_ (%)	−0.73	<0.001	−0.65	<0.001	0.70	<0.001
IQD	−0.57	<0.001	−0.43	0.02	0.43	0.04
IHI	−0.60	<0.001	−0.52	0.003	0.50	0.02

*Note*: Correlations were assessed between pre/post‐bronchodilator lung function and pre/post‐bronchodilator ventilation biomarkers, respectively. All correlations were assessed using Spearman correlation and remained significant after adjusting for multiple comparisons using the Hochberg method.

Abbreviations: FEV1, forced expiratory volume in 1 s; IHI, inhomogeneity index; IQD, interquartile distance; PostBD, post‐bronchodilator; PreBD, pre‐bronchodilator; r, correlation coefficient; RV/TLC, ratio of residual volume to total lung capacity; VDP_FVL_, flow volume loop cross‐correlation ventilation defect percentage; VDP_RVent_, regional ventilation defect percentage.

Post‐bronchodilator, FEV1%_pred_ continued to be significantly correlated with post‐bronchodilator VDP_RVent_ (*ρ* = −0.53, *p* = 0.003), VDP_FVL_ (*ρ* = −0.65, *p* < 0.001), IQD (*ρ* = −0.43, *p* = 0.02), and IHI (*ρ* = −0.52, *p* = 0.003) for all study participants. These correlations remained significant after adjusting for multiple comparisons using the Hochberg method.

The effect of gas trapping (defined as RV/TLC% greater than the predicted upper limit of normal) on ventilation variables was also investigated. Compared to patients without evidence of gas trapping, patients with gas trapping had a significantly higher VDP_RVent_ (26.5 ± 12.3 vs. 11.3 ± 11.3%, *p* = 0.007), VDP_FVL_ (32.3 ± 10.4 vs. 7.7 ± 9.9%, *p* < 0.001), IQD (0.71 ± 0.25 vs. 0.45 ± 0.15, *p* = 0.01), and IHI (0.40 ± 0.11 vs. 0.26 ± 0.09, *p* = 0.003). No significant difference was noted in kurtosis or skewness of ventilation distribution (Table 5).

**TABLE 5:** Effect of gas trapping on ventilation biomarkers.

Variable	Gas trapping	No gas‐trapping	*p* Value
VDP_RVent_ (%)	26.5 ± 12.3	11.3 ± 11.3	0.007
VDP_FVL_ (%)	32.3 ± 10.4	7.7 ± 9.9	<0.001
IQD	0.71 ± 0.25	0.45 ± 0.15	0.01
IHI	0.40 ± 0.11	0.26 ± 0.09	0.003
Kurtosis	2.85 ± 0.84	2.78 ± 0.32	0.80
Skewness	0.26 ± 0.44	0.16 ± 0.27	0.53

Abbreviations: IHI, inhomogeneity index; IQD, interquartile distance; VDP_FVL_, flow volume loop cross‐correlation ventilation defect percentage; VDP_RVent_, regional ventilation defect percentage.

## 4 DISCUSSION

This is the first study to assess changes in ventilation heterogeneity using PREFUL MRI in patients with severe asthma. In this study, we demonstrated the feasibility of this approach, and showed how PREFUL MRI‐derived ventilation biomarkers (VDP and non‐VDP parameters) are (i) significantly worse in asthma patients compared to healthy volunteers; (ii) sensitive to bronchodilator treatment; and (iii) moderately correlate with traditional lung function tests.

All patients recruited in this study had severe asthma as evidence by persistent airflow obstruction on lung function, and high symptom burden despite being on GINA step 4–5 treatment. Additionally, just over 25% of patients required maintenance oral prednisolone. Therefore, it follows that patients should have worse PREFUL MRI derived ventilation biomarkers compared to healthy volunteers. Indeed, this was seen in our study where patients were found to have a significantly higher VDP (RVent and FVL) and worse ventilation heterogeneity parameters when compared to healthy volunteers.

VDP has been commonly used to describe ventilation heterogeneity in the literature. For example, in studies using hyperpolarized gas MRI, the VDP is primarily used as a surrogate for ventilation heterogeneity (Svenningsen et al., 2016; Svenningsen et al., 2018; Svenningsen et al., 2020; Zha et al., 2018) (although ventilation heterogeneity index has been reported in a few studies (Gerald Teague et al., 2021; Hughes et al., 2019; Tzeng et al., 2009)), and represents the percentage of the total lung volume that exhibits no ventilation determined using hierarchical k‐means (Kirby et al., 2012). This method of deriving VDP was utilized by Friedlander et al. in the only other paper investigating PREFUL MRI in severe asthma (Friedlander et al., 2024), and deviates from the original definition used in PREFUL MRI (Pohler et al., 2021).

Apart from VDP, several other measures of ventilation heterogeneity were used in the current study including IQD, IHI, kurtosis, and skewness of ventilation distribution. IQD has previously been reported to be sensitive to changes in ventilation heterogeneity derived using computed tomography in patients with asthma undergoing bronchial thermoplasty (Foo et al., 2024). Similarly, IHI is routinely used to assess ventilation distribution in studies involving electrical impedance tomography (Sang et al., 2020; Yang et al., 2021; Zhao et al., 2009). Among these other measurements, IQD and IHI were felt to be the most sensitive measures of ventilation heterogeneity as they correspond to the dispersion of ventilation, a finding supported in this study. Specifically, IQD and IHI, but not kurtosis or skewness of the ventilation distribution, were observed to be significantly higher in patients with asthma than healthy volunteers, and seen to decrease significantly in patients after bronchodilator therapy. Additionally, significant reductions in VDP_RVent_ were also noted in patients post bronchodilator. Together, these findings highlight the sensitivity of PREFUL derived markers of ventilation heterogeneity to treatment response.

It is worth noting that the VDP used in hyperpolarized gas studies is similar in principle to the VDP_RVent_ and VDP_FVL_ used in PREFUL MRI, in that all three metrics describe “defects” based on values below a pre‐defined threshold. Unlike IQD and IHI, one shortcoming of this approach is the over‐emphasis on the “defects” with little consideration for the remaining spectrum of ventilation (hypo‐, normal and hyper‐ventilated areas). Airway remodeling has generally been viewed as a detrimental manifestation contributing to a progressive, irreversible decline in lung function. In contrast to these concerns, an alternative hypothesis may be that stiffening of airways with remodeling is a compensatory response which maintains airway patency by limiting effects of repeated bronchoconstriction (McParland et al., 2003). Hence, distinguishing between complete closure and constricted but patent airways with a spectrum of ventilation, rather than just defects, may provide further insights into the contribution of airway remodeling in asthma (Nilsen et al., 2021).

The VDP (RVent and FVL) results shown here are largely consistent with those reported by others using similar PREFUL methodology. For instance, healthy volunteers were found to have a VDP of just under 2% in this study, well within the 8%–10% upper limit described in the literature (Glandorf et al., 2020; Moher Alsady, Ruschepaul, et al., 2024; Pohler et al., 2021). Likewise, an average VDP of ~20% was observed in those with severe asthma, in line with the 20%–40% described in patients with other forms of airway disease (chronic obstructive pulmonary disease and cystic fibrosis) (Glandorf et al., 2020; Marshall et al., 2023; Moher Alsady et al., 2019; Moher Alsady, Ruschepaul, et al., 2024; Pohler et al., 2021; Voskrebenzev et al., 2022). We also report IQD and IHI values approximately twice as high in patients with severe asthma than in healthy volunteers. As IQD and IHI are being used for the first time in the evaluation of ventilation heterogeneity in PREFUL MRI, no prior data exist for comparison. Nonetheless, the shape of the ventilation histogram of the pooled healthy volunteer data is consistent with our understanding of lung physiology inasmuch as some degree of ventilation heterogeneity is to be expected even in normal healthy lungs due to the regional differences in ventilation arising from the effects of gravity, airway resistance, and compliance (West & Luks, 2021). The striking visual differences in ventilation histograms as depicted in Figure 1a between patients and healthy volunteers provide further support for the validity of IQD and IHI as indicators of ventilation heterogeneity.

The normalized pooled histogram of the ventilation distribution in healthy volunteers appeared to be slightly wider and flatter post‐bronchodilator, suggesting an increase in ventilation heterogeneity although no significant change was observed in any of the PREFUL derived biomarkers. Although counter‐initiative, this observation may be explained by the loss of the protective effect of the airway smooth muscle in preserving small airway patency post‐bronchodilator (Bosse, 2025). For example, Crawford et al. found that relaxing the airway smooth muscle in healthy subjects by obliterating the bronchomotor tone with intravenous atropine resulted in a 37% increase in ventilation heterogeneity measured by multiple breath nitrogen washout (Crawford et al., 1987). A subsequent study by Kelly et al. substantiated this finding by showing that residual volume and gas‐trapping significantly increased post‐bronchodilator in healthy subjects, with the latter indicative of air trapping due to closed airways (Kelly et al., 2012). Due to the small sample size in the current study, these observations would need to be confirmed in future studies.

Significant correlations were found between pre‐bronchodilator FEV1%_pred_ and baseline VDP_RVent_, VDP_FVL_, IQD, and IHI, suggesting that patients who were the most obstructed at baseline also had worse ventilation biomarkers. Although similar correlations were noticed between post‐bronchodilator FEV1%_pred_ and post‐bronchodilator VDP_RVent_, VDP_FVL_, IQD, and IHI, the strength of these correlations was weaker. Given the relative changes in FEV1%_pred_ and ventilation biomarkers post‐bronchodilator were sizable (e.g., mean FEV1%_pred_ increased by 13% while mean RVent decreased by 26%), the weaker correlations observed post‐bronchodilator may be explained by the differences in how PREFUL MRI and FEV1 capture physiological change post‐bronchodilator.

There are several limitations that need to be acknowledged. Firstly, although we report compelling evidence for the use of PREFUL MRI in the assessment of ventilation heterogeneity in severe asthma, these results need to be confirmed in larger studies including subjects with varying asthma severity and eventually in other pulmonary conditions. Secondly, the current study involved predominantly female subjects, and future trials should incorporate sex and gender considerations into the study design. Thirdly, healthy volunteers were younger and had a lower BMI than patients with asthma. Given age and BMI are factors known to influence lung function (Roman et al., 2016), groups should be matched for these factors in future studies. Fourthly, only single‐slice 2D PREFUL data was acquired and analyzed for this study as 3D PREFUL sequences have yet to be implemented at our centre (Klimes, Voskrebenzev, Gutberlet, Kern, et al., 2021; Klimes, Voskrebenzev, Gutberlet, Obert, et al., 2021). Despite this, our single‐slice approach is consistent with that described by many others in the field (Friedlander et al., 2024; Munidasa et al., 2021; Svenningsen et al., 2016; Svenningsen et al., 2018; Wang et al., 2022). Lastly, correlation with advanced pulmonary function tests such as multiple breath nitrogen washout and oscillometry should also be considered in future studies.

## 5 CONCLUSION

In conclusion, we have demonstrated the feasibility of assessing ventilation heterogeneity in patients with severe asthma using PREFUL MRI. We have shown how increased ventilation heterogeneity correlates with the severity of airflow obstruction and reduces in response to bronchodilator therapy. Given the recent interest in ventilation heterogeneity as a treatable trait, these findings expand the role of PREFUL MRI to the study of other pulmonary diseases where ventilation heterogeneity is thought to play a key pathophysiological role.

## AUTHOR CONTRIBUTIONS

CF: conception, data acquisition, analysis and interpretation, and drafted and revised manuscript. DL: conception, data acquisition and interpretation, and revision of manuscript. GD and PN: conception, data analysis and interpretation, and revision of manuscript. BT and FT: conception, data interpretation, and revision of manuscript. All authors approved the final version of the manuscript.

## FUNDING INFORMATION

This work was supported by the National Australian Health and Medical Research Council (NHMRC Grant number APP1180854), for which P.N is the grant holder. C.F is the recipient of a Monash University postgraduate scholarship.

## CONFLICT OF INTEREST STATEMENT

The authors declare that they have no competing interests.

## ETHICS STATEMENT

The study was approved by the Eastern Health Human Research Ethics Committee and conducted in accordance with The Code of Ethics of the World Medical Association (Declaration of Helsinki).

## Data Availability

De‐identified data is available from the corresponding author upon reasonable request and subject to a formal data‐sharing agreement.
